# A Class of Quadratic Polynomial Chaotic Maps and Their Fixed Points Analysis

**DOI:** 10.3390/e21070658

**Published:** 2019-07-04

**Authors:** Chuanfu Wang, Qun Ding

**Affiliations:** Electronic Engineering College, Heilongjiang University, Harbin 150080, China

**Keywords:** hidden attractors, polynomial chaotic maps, amplitude control, approximate entropy

## Abstract

When chaotic systems are used in different practical applications, such as chaotic secure communication and chaotic pseudorandom sequence generators, a large number of chaotic systems are strongly required. However, for a lack of a systematic construction theory, the construction of chaotic systems mainly depends on the exhaustive search of systematic parameters or initial values, especially for a class of dynamical systems with hidden chaotic attractors. In this paper, a class of quadratic polynomial chaotic maps is studied, and a general method for constructing quadratic polynomial chaotic maps is proposed. The proposed polynomial chaotic maps satisfy the Li–Yorke definition of chaos. This method can accurately control the amplitude of chaotic time series. Through the existence and stability analysis of fixed points, we proved that such class quadratic polynomial maps cannot have hidden chaotic attractors.

## 1. Introduction

When chaotic systems are used in different practical applications, such as chaotic secure communication and chaotic pseudorandom sequence generators, a large number of chaotic systems are strongly required. The traditional design schemes of classical chaotic systems have no definite mechanics and specific design steps, so it is difficult to construct a large number of new chaotic systems. With the development of the computer, the exhaustive search method has become the mainstream method for finding new chaotic systems. In 2000, Sprott searched many new chaotic maps by the exhaustive search method [1]. However, the efficiency of the exhaustive search is very low because the search range of parameters is too large. In addition, the initial values may be not in the basin of the attractors, so we will always neglect massive new chaotic maps. For the exhaustive search method, the probability of finding new chaotic maps was 2.3%. In addition to the exhaustive search method, a small number of chaotic system design algorithms have also been proposed, owing to the study of the Jacobian matrix of the chaotic system. In 1999, Chen and Lai proposed the Chen–Lai algorithm to control the Jacobian matrix of a chaotic system [2]. Since the Jacobian matrix of a chaotic system is closely related to Lyapunov exponents, the Chen–Lai algorithm can be used as the principle to construct new discrete chaotic maps. Based on the Chen–Lai algorithm, some new general design methods for constructing chaotic systems were proposed [3,4,5,6]. These design methods all adjust the Lyapunov exponent by using the feedback function to control the Jacobian matrix. In 2018, a general method of reconstructing new chaotic systems by using predetermined positive Lyapunov exponents was also proposed [7]. In addition, some new chaotic systems can also be designed by the method of parameter perturbation and coupling [8,9,10,11]. However, these methods can only work in certain chaotic systems, and cannot be used as a general method for designing new chaotic systems. 

Compared with traditional chaotic systems, there are some special chaotic systems, which are excited from stable fixed points or have no fixed points. The attractors of those special chaotic systems are called hidden attractors. Hidden attractors imply that the basin of attraction does not contain neighborhoods of fixed points. Since the general construction method of traditional chaotic systems cannot effectively analyze the existence and stability of fixed points, the exhaustive search is still the main way to find new chaotic systems with hidden chaotic attractors. For the randomness of the exhaustive search method, we can only find several special chaotic systems with hidden attractors randomly, and cannot systematically analyze the existence of chaotic hidden attractors in a wide class of dynamical systems, especially for discrete chaotic systems. At present, the research on constructing discrete chaotic systems with hidden attractors mainly focuses on the passive brute force parametric investigation or initial values investigation in a class of two-dimensional polynomial chaotic maps or three-dimensional polynomial chaotic maps [12,13]. In addition, few special chaotic maps without fixed points based on the logistic map and Arnold’s cat map have also been proposed [14,15]. For the lack of a systematic theory on fixed points, the existing general method of constructing new traditional chaotic maps and the analysis of chaotic hidden attractors cannot be combined organically.

The chaotization of the dynamical systems methods based on the geometry is very rare. The period-three points play a vital role in the chaotic map because Li and Yorke have shown that period-three implies chaos, and proposed the famous period-three theorem [16]. In the first 11 years, Sharkovskii also proposed the Sharkovskii theorem on period-three points [17]. According to the period-three theorem, we propose a general method to construct quadratic polynomial chaotic maps, and systematically analyze the existence of hidden attractors in such a class of quadratic polynomial chaotic maps. The general method proposed in this paper overcomes the defect of the low efficiency of finding new chaotic maps by exhaustive search and poor amplitude control ability by the existing general construction method of traditional chaotic systems. According to the period-three theorem, the proposed chaotic systems satisfy the Li–Yorke definition of chaos. Through the existence and stability analysis of fixed points, we prove that such a class of quadratic polynomial maps cannot have hidden chaotic attractors.

In Section 2, the mechanism of constructing quadratic polynomial chaotic maps with controllable amplitude is analyzed. It includes the reconstruction of the quadric polynomial chaotic map, fixed points analysis, and amplitude control analysis. In Section 3, three different concrete examples are given. In Section 4, the mechanism of constructing high-degree polynomial chaotic maps with controllable amplitude is proposed. Conclusions are made in Section 5. 

## 2. The Mechanism of Constructing Quadratic Polynomial Chaotic Maps with Controllable Amplitude

According to the period-three theorem, the mechanism of a class of quadratic polynomial chaotic maps is proposed in this section, and the existence and stability of their fixed points are also analyzed.

### 2.1. Constructing Quadratic Polynomial Chaotic Maps

In this section, the quadric polynomial maps are taken into consideration. The general quadric polynomial function is defined as:

(1) $$f_{2}(x)=ax^{2}+bx+d,$$

where a, b, and d are all coefficients. According to the existence of period-three points, three equations can be obtained:

(2) $$\{\begin{array}{c} f_{2}(A)=aA^{2}+bA+d=B\\ f_{2}(B)=aB^{2}+bB+d=D\\ f_{2}(D)=aD^{2}+bD+d=A \end{array},$$

where A, B, D are three arbitrary numbers, and A<B<D. From Equation (2), we can know that numbers A, B, and D are period-three points separately, and they are in the same period orbit. The three coefficients of the function $f_{2}(x)$ can be represented by period-three points through solving Equation (2):

(3) $$a=\frac{{\left(B-D\right)}^{2}-(D-A)(A-B)}{\left(A-B)(B-D)(A-D\right)};$$

(4) $$b=\frac{\left(B-D)(B^{2}-D^{2})-(D-A)(A^{2}-B^{2}\right)}{\left(A-B)(B-D)(D-A\right)}.$$

The coefficient d can be obtained by putting a, b into any one of three equations of Equation (2). For example,

(5) $$d=B-aA^{2}-bA.$$

Bringing a, b, and d into Equation (1), we can construct a discrete quadric polynomial chaotic map:

(6) $$x(n+1)=a{\left[x(n)\right]}^{2}+bx(n)+d.$$

According to the period-three theorem, the chaotic system proposed in this paper satisfies the Li–Yorke definition of chaos. Since the reconstructed quadric polynomial chaotic map has period-three points, we can further limit the range of parameters a, b, and d.

**Theorem** **1.** *For*0<A<B<D*, if map (6) is a chaotic map, then*a<0*,*b>2*, and*d<0.

**Proof:**  when 0<A<B<D, we can know that A−B<0, B−D<0, and A−D<0. For the parameter a in map (6), the denominator (A−B)(B−D)(A−D)<0, and (D−A)(A−B)<0. The numerator in Equation (3) is greater than zero, that is ${\left(B-D\right)}^{2}-(D-A)(A-B)>0$. Since the denominator is less than zero and numerator is greater than zero, the parameter a in map (6) is less than zero.

For the parameter b in map (6), we can obtain that:

$$\begin{array}{l} b-2=\frac{\left(B-D)(B^{2}-D^{2})-(D-A)(A^{2}-B^{2})-2(A-B)(B-D)(D-A\right)}{\left(A-B)(B-D)(D-A\right)}\\ \;=\frac{\left(B-D)(B^{2}-D^{2})-(A-B)(B-D)(D-A)-(D-A)(A^{2}-B^{2})-(A-B)(B-D)(D-A\right)}{\left(A-B)(B-D)(D-A\right)}\\ \;=\frac{\left(D-B)[D^{2}-B^{2}-(B-A)(D-A)]+(D-A)[B^{2}-A^{2}-(B-A)(D-B)\right]}{\left(A-B)(B-D)(D-A\right)}\\ >\frac{\left(D-B)[D^{2}-B^{2}-(B-A)(D-A)]+(D-B)[B^{2}-A^{2}-(B-A)(D-B)\right]}{\left(A-B)(B-D)(D-A\right)}\\ =\frac{\left(D^{2}-2BD+2AD+B^{2}-2A^{2}\right)}{\left(B-A)(D-A\right)}\\ =\frac{\left[{\left(D+B\right)}^{2}+2A(D-A)\right]}{\left(B-A)(D-A\right)}>0. \end{array}$$

For the parameter d in map (6), we bring Equation (3) and Equation (4) into Equation (5). Then, we also can obtain that:

$$\begin{array}{l} \;d=B-aA^{2}-bA\\ \;<A-aA^{2}-bA\\ \;=-A(aA+b-1)\\ \;=(-A)\frac{A{\left(B-D\right)}^{2}-A(D-A)(A-B)+(B-D)(B^{2}-D^{2})-(D-A)(A^{2}-B^{2})-(A-B)(B-D)(D-A)}{\left(A-B)(B-D)(D-A\right)}\\ =(-A)\frac{\left(D-B)(D-B)(A+D+B)+(D-A)(B-A)(2B+2A-D\right)}{\left(A-B)(B-D)(D-A\right)}\\ <(-A)\frac{\left(D-B)(2AD+D^{2}-BD-AB+B^{2}-2A\right)}{\left(A-B)(B-D)(D-A\right)}\\ =(-A)\frac{\left(D-B)[D(D-B)+B(B-A)+2A(D-A)\right]}{\left(A-B)(B-D)(D-A\right)}<0. \end{array}$$

### 2.2. Fixed Points Analysis

Then, we analyze the fixed points of map (6). Let x(n+1)=x(n), map (6) is changed into:

(7) $$a{\left[x(n)\right]}^{2}+(b-1)x(n)+d=0.$$

For the quadric polynomial equation, the roots can be represented by the coefficients. According to quadratic formula, the roots of Equation (7) are:

(8) $$\{\begin{array}{c} x_{1}=\frac{-(b-1)+\sqrt{{\left(b-1\right)}^{2}-4ad}}{2a}\\ x_{2}=\frac{-(b-1)-\sqrt{{\left(b-1\right)}^{2}-4ad}}{2a} \end{array}.$$

The term $\Delta ={\left(b-1\right)}^{2}-4ad$ of Equation (7) determines the number of fixed points. According to the polarity of term $\Delta ={\left(b-1\right)}^{2}-4ad$, the number of roots can be divided into three different situations. When $\Delta ={\left(b-1\right)}^{2}-4ad>0$, Equation (6) has two different fixed points. When $\Delta ={\left(b-1\right)}^{2}-4ad=0$, Equation (6) has one fixed point. When $\Delta ={\left(b-1\right)}^{2}-4ad<0$, Equation (6) also has no fixed point. In addition, the first order derivative of map (6) determines the stability of the fixed points. The first order derivative of map (6) is:

(9) J(x)=2ax+b.

For a fixed point $x_{1}$, if $\left|J(x_{1})\right|<1$, it is a stable fixed point. If $\left|J(x_{1})\right|>1$, $x_{1}$ is an unstable fixed point.

#### 2.2.1. Two Fixed Points

**Theorem** **2.** *For* 0<A<B<D *, if map (6) has a chaotic attractor,* $\Delta ={\left(b-1\right)}^{2}-4ad>0$ *and it must have two different fixed points.*

**Proof**: If chaotic map (6) has two different fixed points, then $\Delta ={\left(b-1\right)}^{2}-4ad>0$, that is ${\left(b-1\right)}^{2}-4ad>0$. If ${\left(b-1\right)}^{2}-4ad>1$, then $b^{2}+2b-4ad>0$. Suppose ${\left(b-1\right)}^{2}-4ad>1$, it is also equal to $b^{2}-2ad+2(b-ad)>0$. According to Theorem 1, b>2. Therefore, $b^{2}-2ad+2(b-ad)>4(b-ad)$. Then, we just need to prove b−ad>0. Combining with Equation (4), we can obtain that:

$$\begin{array}{c} b-ad=b+a^{2}A^{2}+abA-aB\\ \;>b+a^{2}A^{2}+abA-aA\\ >a^{2}A^{2}+(b-1)aA+b+\frac{{\left(b-1\right)}^{2}}{4}-\frac{{\left(b-1\right)}^{2}}{4}\\ ={\left(aA+\frac{b-1}{2}\right)}^{2}-\frac{{\left(b+1\right)}^{2}}{4}=(aA+b)(aA+1). \end{array}$$

For (aA+b−1), we also can obtain that:

$$\begin{array}{l} (aA+b-1)=\frac{A{\left(B-D\right)}^{2}-A(D-A)(A-B)+(B-D)(B^{2}-D^{2})-(D-A)(A^{2}-B^{2})-(A-B)(B-D)(D-A)}{\left(A-B)(B-D)(D-A\right)}\\ =\frac{\left(D-B)(D-B)(A+D+B)+(D-A)(B-A)(2B+2A-D\right)}{\left(A-B)(B-D)(D-A\right)}\\ >\frac{\left(D-B)(2AD+D^{2}-BD-AB+B^{2}-2A\right)}{\left(A-B)(B-D)(D-A\right)}\\ =\frac{\left(D-B)[D(D-B)+B(B-A)+2A(D-A)\right]}{\left(A-B)(B-D)(D-A\right)}>0. \end{array}$$

According to Theorem 1, we know that b>2. Therefore, aA+1>0, and aA+b>0. If map (6) is a chaotic map with positive period-three points, then it must have two different fixed points. Then, we consider the stability of two different fixed points. According to Equation (8), we bring $x_{1}$ and $x_{2}$ into Equation (9). Then, we obtain Equation (10):

(10) $$\{\begin{array}{c} J(x_{1})=1+\sqrt{{\left(b-1\right)}^{2}-4ad}\\ J(x_{2})=1-\sqrt{{\left(b-1\right)}^{2}-4ad} \end{array}.$$

Since $\Delta ={\left(b-1\right)}^{2}-4ad>0$, $J(x_{1})=1+\sqrt{{\left(b-1\right)}^{2}-4ad}>1$, it is an unstable fixed point in map (6). Therefore, such quadratic polynomial chaotic maps cannot have hidden chaotic attractors excited from stable fixed points.

#### 2.2.2. One Fixed Point

According to Theorem 2, ${\left(b-1\right)}^{2}-4ad>0$. When map (6) is the chaotic map with positive period-three points, it cannot have one fixed point. Therefore, such quadratic polynomial chaotic maps cannot have hidden chaotic attractors with one stable fixed point.

#### 2.2.3. No Fixed Points

According to Theorem 2, ${\left(b-1\right)}^{2}-4ad>0$. When map (6) is the chaotic map with positive period-three points, it must have two different fixed points. Therefore, such quadratic polynomial chaotic maps cannot have hidden chaotic attractors without fixed points. Through the existence and stability analysis of fixed points, we find that such quadratic polynomial maps with positive period-three points cannot have hidden chaotic attractors.

### 2.3. Amplitude Analysis

According to the period-three theorem, we can choose the appropriate period-three points to control the amplitude of the reconstructed chaotic maps for satisfying practical engineering needs. For the existing chaotic maps, we can indirectly control the amplitude of the chaotic time series by controlling the period-three points. Maybe we don’t know exactly what the period-three points of a chaotic map are, but we can control the amplitude of chaotic time series by changing the period-three points. Suppose the new period-three points are $A{}^{\prime }=MA$,$B{}^{\prime }=MB$, and $D{}^{\prime }=MD$. Bringing them into Equation (3), Equation (4), and Equation (5), we can obtain $a{}^{\prime }=aM$, $b=b{}^{\prime }$, and $d{}^{\prime }=\frac{d}{M}$.

### 2.4. Concrete Scheme

In this section, two concrete schemes are given, one is to construct a new quadratic polynomial chaotic map with controllable amplitude, the other is to control the amplitude of the existing quadratic polynomial chaotic map.

The concrete method for constructing a new quadratic polynomial chaotic map with controllable amplitude is shown below:

**Step 1.** Give three different number A, B, and D, such that A<B<D.

**Step 2.** Bringing A, B, and D into Equation (3), Equation (4), and Equation (5), we can obtain parameters a, b, and d.

**Step 3.** Finally, we can reconstruct a new quadratic polynomial chaotic map $x(n+1)=a{\left[x(n)\right]}^{2}+bx(n)+d$.

The concrete method for controlling the amplitude of the existing quadratic polynomial chaotic map is shown below:

**Step 1.** Choose an appropriate factor M. If M>1, the chaotic time series will increase M times. If M<1, the chaotic time series will reduce M times.

**Step 2.** we can obtain a new quadratic polynomial chaotic $x(n+1)=aM{\left[x(n)\right]}^{2}+bx(n)+\frac{d}{M}$ with appropriate amplitude.

## 3. Examples and Simulations

### 3.1. Constructing New Quadratic Polynomial Chaotic Maps

Give different numbers A=1, B=2, D=3. Then, bring them into Equation (3), Equation (4), and Equation (5). We can obtain the coefficients a=−1.5, b=5.5, and d=−2. The reconstructed new quadratic polynomial chaotic map is:

(11) $$x(n+1)=-1.5{\left[x(n)\right]}^{2}+5.5x(n)-2.$$

Since a=−1.5<0, b=5.5>2, and d=−2<0, the coefficients of chaotic map (11) strictly satisfy Theorem 1. Let x(n+1)=x(n), chaotic map (11) is changed into:

(12) $$-1.5{\left[x(n)\right]}^{2}+4.5x(n)-2=0.$$

According to the relationship between roots and coefficients, we can obtain two different roots of map (12):

(13) $$\{\begin{array}{c} x_{1}=2.4574\\ x_{2}=0.5426 \end{array}.$$

The first order derivative of map (11) is,

(14) J(x)=−3x+5.5.

After bringing two different fixed points into Equation (14), we obtain the following:

$$\{\begin{array}{c} J(2.4571)=-1.8723\\ J(0.5426)=3.8723 \end{array}$$

**Figure 1:**
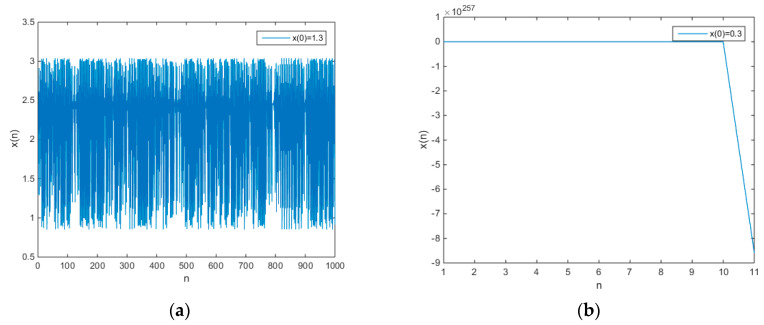

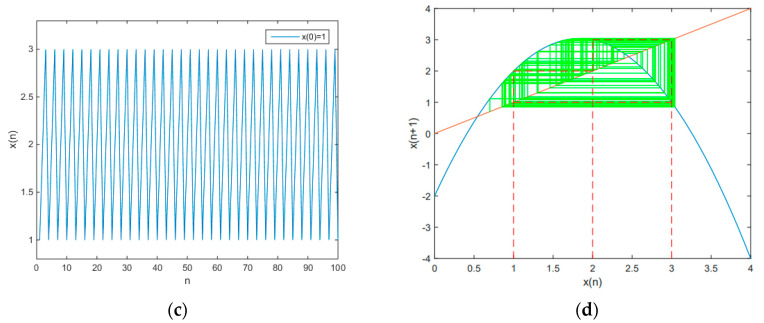
The output chaotic time series under a=−1.5, b=5.5, and d=−2, and iterative route diagram (**a**) initial value x(0)=1.3, (**b**) initial value x(0)=0.3, (**c**) initial value x(0)=1, (**d**) iterative route diagram.

Since |J(2.4571)|=1.8723>1, the fixed point $x_{1}$ is an unstable fixed point. Since J(0.5426)=3.8723>1, the fixed point $x_{2}$ is also an unstable fixed point. Through the Jacobian method, the Lyapunov exponent of chaotic map (11) is 0.4383. The output time series of chaotic map (11) is shown in Figure 1.

From Figure 1a, when x(0)=1.3, it is obvious that the output time series of chaotic map (11) shows chaotic behavior. Since A=1<x(0)=1.3<B=2, the initial value x(0)=1.3 will lead to a chaotic orbit. From Figure 1b, when x(0)=0.3, the amplitude of output time series is unbounded. Since x(0)=0.3<<A=1, the point x(0)=0.3 is not in the basin of chaotic attractors. Therefore, the initial value x(0)=0.3 will lead to an orbit with unbounded amplitude. From Figure 1c, when x(0)=1, the output time series is periodic because the initial value is exactly in the period-three orbit (x(0)=A=1). The iterative route of chaotic map (11) is shown in Figure 1d. The iterative route (red stair step) of the period-three points “frames” most of the iterative routes (green stair step). Therefore, as long as the initial value is in an open interval consisting of any two points of period-three points, then it is likely to be in a basin of chaotic attractors. The initial value has a very important influence on dynamical systems. Different initial values may produce different behavior. Figure 1a shows a chaotic behavior, Figure 1b shows an unbounded behavior, and Figure 1c shows a periodic behavior. As early as 2000, Sprott explicitly stated the impact of initial values on finding chaotic systems by exhaustive search [1]. For the general quadric polynomial maps, it has three parameters. After adding the initial value, there is a total of four variants. In those four variants, we need to select some specific values, which lead to chaotic behavior in a large value space. Sprott found that the probability of finding new chaotic maps was 2.3% (of the 15,625 combinations of coefficient, exactly 364 can generate strange chaotic attractors) [1]. The exhaustive search for four variants is not a difficult task, but for a large search scope, it may be tough work to find more new chaotic maps. However, the method proposed in this paper can overcome this shortcoming very well. According to the period-three theorem, the chaotic system proposed in this paper satisfies the Li–Yorke definition of chaos. When a chaotic system satisfies the Li–Yorke definition of chaos, the chaotic behavior may not be observed because the Lebesgue integral of the chaotic domain may be zero. However, people are interested in a situation where chaotic behavior can be observed. Therefore, we just need to select an appropriate initial value, such as A<x(0)<B, and observe the output time series. Compared with exhaustive search, the probability of finding a new chaotic system by using the proposed new method is far greater than 2.3%.

### 3.2. Amplitude Control

#### 3.2.1. Amplitude Control of New Chaotic Maps

In this section, we propose some simple models to illustrate the control of chaotic time series by period-three points. Let A=t, B=t+w, and D=t+kw. For different parameters t, w, and k, different quadric polynomial maps can be reconstructed. In Equation (15), we propose four different new chaotic maps.

(15) $$\{\begin{array}{ll} x(n+1)=-3{\left[x(n)\right]}^{2}+3.46x(n)+0.18,t=0.16,w=0.5,k=2, & \;(15a)\\ x(n+1)=-3{\left[x(n)\right]}^{2}+6.34x(n)-1.69,t=0.64,w=0.5,k=2, & \;(15b)\\ x(n+1)=-0.3{\left[x(n)\right]}^{2}+2.6x(n)+4.75,t=0.16,w=5,k=2, & \;(15c)\\ x(n+1)=-150{\left[x(n)\right]}^{2}+194.5x(n)-62.39,t=0.64,w=0.01,k=2, & \;(15d) \end{array}$$

**Figure 2:**
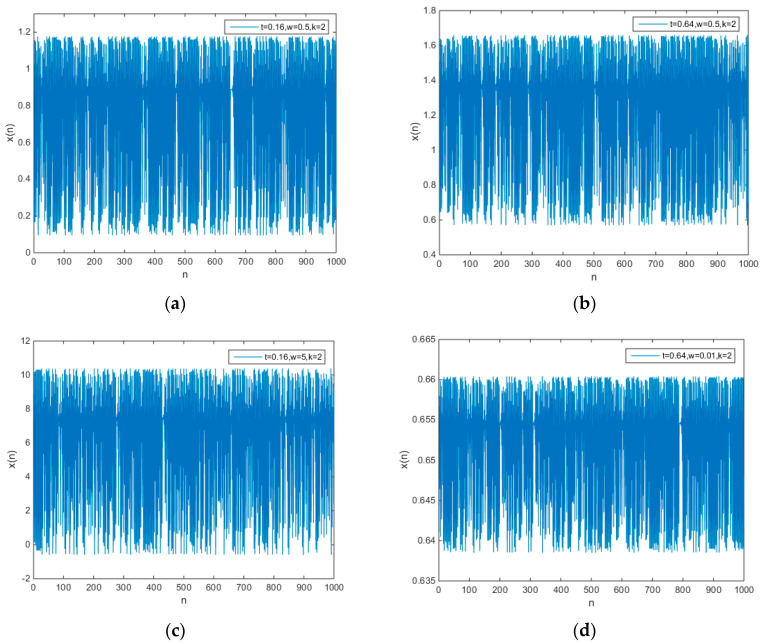
The output chaotic time series of chaotic maps (15) (**a**) t=0.16,w=0.5,k=2, (**b**) t=0.64,w=0.5,k=2, (**c**) t=0.16,w=5,k=2, (**d**) t=0.64,w=0.01,k=2.

The initial values of all the chaotic maps in Equation (15) are set as t+0.001, and the chaotic time series of chaotic maps are shown in Figure 2.

When the parameters t, w, and k are changed, the chaotic time series also is changed. From Figure 2, we can know that the method proposed in this paper can control the amplitude of chaotic time series well. However, for other methods [3,4,5,6,7,8,9,10,11], neither of them can control the amplitude of chaotic time series.

#### 3.2.2. Amplitude Control of the Existing Chaotic Map

The method proposed in this paper can also be used to control the amplitude of existing classical chaotic maps. For example, classical quadratic logistic chaotic maps [18] are defined as

(16) $$x(n+1)=4x(n)-4{\left[x(n)\right]}^{2},x(n)\in [0.1].$$

When factor M=0.25,0.5,2,4, the new models based on map (15) can be obtained:

(17) $$\{\begin{array}{ll} x(n+1)=4x(n)-{\left[x(n)\right]}^{2},M=4, & \;(17a)\\ x(n+1)=4x(n)-2{\left[x(n)\right]}^{2},M=2, & \;(17b)\\ x(n+1)=4x(n)-8{\left[x(n)\right]}^{2},M=0.5, & \;(17c)\\ x(n+1)=4x(n)-16{\left[x(n)\right]}^{2},M=0.25, & \;(17d) \end{array}.$$

**Figure 3:**
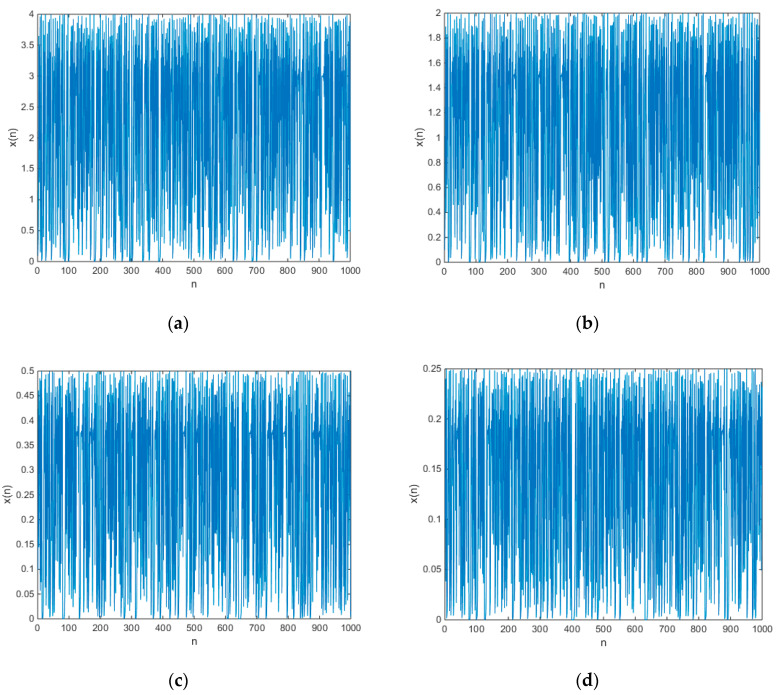
The output chaotic time series of chaotic maps (17) (**a**) M=4, (**b**) M=2, (**c**) M=0.5, (**d**) M=0.25.

The initial values of all the chaotic maps in Equation (17) are set as 0.1, and the chaotic time series are shown in Figure 3.

The factor M obviously controls the amplitude of the output time series of chaotic map (17). Although we do not know the exact period-three points of logistic map, it can be sure that the period-three points must satisfy Equation (3), Equation (4), and Equation (5). Since the factor M changes the period-three points of logistic map, it indirectly controls the amplitude of logistic map.

### 3.3. Approximate Entropy Analysis

In statistics, approximate entropy (ApEn) is a technique used to quantify the amount of regularity and unpredictability of fluctuations over time series data. The main idea of approximate entropy is to use a non-negative value to quantify the complexity and irregularity of the time series, and the value increases with increased sequence complexity. The calculation process of the approximate entropy is shown as follows [15]:

1. Suppose the initial data is the sequence x(1), x(2), …x(N), and then divide them into m-dimensional vectors:
  (18) X(i)=[x(i),x(i+1),…,x(i+m−1)], where i=1,2,3…N−m+1;
2. The distance between x(i) and x(j) is defined as:
  (19) $$d(i,j)=\underset{k=1-m-1}{\max}[\left|x(i+k)-x(j+k)\right|];$$
3. Setting a threshold value r (r>0), for each i, we can obtain the statistics of d(i,j):
  (20) $$C_{i}{}^{m}(r)=\frac{1}{N-m+1}Sum\{d(i,j)<r\};$$
4. The mean of the logarithm of $C_{i}{}^{m}(r)$ is written as $\phi^{m}(r)$ and can be calculated by:
  (21) $$\phi^{m}(r)=\frac{1}{N-m+1}\sum_{i=1}^{N-m+1}\ln C_{i}{}^{m}(r);$$
5. Changing dimension and repeating step 1 to step 4, we can obtain the approximate entropy:
  (22) $$ApEn(m,r)=\underset{N\to \infty }{\lim}[\phi^{m}(r)-\phi^{m+1}(r)].$$

However, in practical applications, the length of the data sequence is bounded. Therefore, the approximate entropy algorithm is changed into:

(23) $$ApEn(m,r,N)=[\phi^{m}(r)-\phi^{m+1}(r)].$$

Pincus found that there is a minimal dependency between ApEn and N when m=2 and r∈[0.1SD(x),0.2SD(x)] [15]. SD(x) is the standard deviation of x(n). The complexity of the output time series of chaotic maps (11), (15), (16), and (17) are tested by the approximate entropy algorithm, and the consequence shows that the output time series of Equation (17d) has a higher complexity. The specific results are shown in Table 1.

## 4. Constructions of High-Degree Polynomial Chaotic Maps

The proposed method can also be applied to the construction of high-degree polynomial chaotic maps. For general high-degree polynomial function,

(24) $$f_{k}(x)=a_{\left(0\right)}x^{k}+a_{\left(1\right)}x^{k-1}+\ldots a_{\left(k-1\right)}x+a_{\left(k\right)},\;k\geq 3$$

For three different numbers of A, B, and D, three equations can be obtained by the existence of period-three points:

(25) $$\{\begin{array}{l} f_{k}(A)=a_{\left(0\right)}A^{k}+a_{\left(1\right)}A^{k-1}+\ldots a_{\left(k-1\right)}A+a_{\left(k\right)}=B\\ f_{k}(B)=a_{\left(0\right)}B^{k}+a_{\left(1\right)}B^{k-1}+\ldots a_{\left(k-1\right)}B+a_{\left(k\right)}=D\\ f_{k}(D)=a_{\left(0\right)}D^{k}+a_{\left(1\right)}D^{k-1}+\ldots a_{\left(k-1\right)}D+a_{\left(k\right)}=A \end{array}.$$

There are k coefficients and three equations in Equation (25). Therefore, Equation (25) could have more than one solution. Suppose $a_{\left(0\right)},a_{\left(1\right)}\ldots a_{\left(k-3\right)}$ are all constants, and $Q_{k}(x)=a_{\left(0\right)}x^{k}+a_{\left(1\right)}x^{k-1}+\ldots +a_{\left(k-3\right)}x^{3}$. Combining equation $Q_{k}(x)$, Equation (25) is changed to Equation (26):

(26) $$\{\begin{array}{l} Q_{k}(A)+a_{\left(k-2\right)}A^{2}+a_{\left(k-1\right)}A+a_{\left(k\right)}=B\\ Q_{k}(B)+a_{\left(k-2\right)}B^{2}+a_{\left(k-1\right)}B+a_{\left(k\right)}=D\\ Q_{k}(D)+a_{\left(k-2\right)}D^{2}+a_{\left(k-1\right)}D+a_{\left(k\right)}=A \end{array}.$$

The method of solving solution of Equation (26) is similar to that of Equation (2).

(27) $$a_{\left(k-2\right)}=\frac{{\left(B-D\right)}^{2}-(D-A)(A-B)}{\left(A^{2}-B^{2})(B-D)-(B^{2}-D^{2})(A-B\right)}-\frac{\left[(Q_{k}(A)-Q_{k}(B))(B-D)-(Q_{k}(B)-Q_{k}(D))(A-B)\right]}{\left(A^{2}-B^{2})(B-D)-(B^{2}-D^{2})(A-B\right)}$$

(28) $$a_{\left(k-1\right)}=\frac{\left(B-D)(B^{2}-D^{2})-(D-A)(A^{2}-B^{2})-[(Q_{k}(A)-Q_{k}(B))(B^{2}-D^{2})-(Q_{k}(B)-Q_{k}(D))(A^{2}-B^{2})\right]}{\left(A-B)(B^{2}-D^{2})-(B-D)(A^{2}-B^{2}\right)}$$

Coefficient $a_{\left(k\right)}$ can be obtained by putting $a_{\left(k\right)}$, $a_{\left(k-1\right)}$ into any one of the three equations of Equation (26).

For example,

(29) $$a_{\left(k\right)}=B-a_{\left(k-2\right)}A^{2}-a_{\left(k-1\right)}A-Q_{k}(A).$$

Then, the high-degree polynomial chaotic maps can be reconstructed:

(30) $$x(n+1)=a_{\left(0\right)}{\left[x(n)\right]}^{k}+a_{\left(1\right)}{\left[x(n)\right]}^{k-1}+\ldots +a_{\left(k-1\right)}x(n)+a_{\left(k\right)}.$$

According to the period-three theorem, the chaotic system proposed in this paper satisfies the Li–Yorke definition of chaos. The concrete method for constructing a new high-degree polynomial chaotic map with controllable amplitude is shown below:

**Step 1.** Give three different numbers for A, B, and D, such that A<B<D, and ensure k−3 number $a_{\left(0\right)},a_{\left(1\right)}\ldots a_{\left(k-3\right)}$ are not equal to the zero at the same time;

**Step 2.** Bringing A, B, D, and $a_{\left(0\right)},a_{\left(1\right)}\ldots a_{\left(k-3\right)}$ into Equation (27), Equation (28), and Equation (29), we can obtain parameters a, b, and d;

**Step 3.** Finally, we can reconstruct a new high-degree polynomial chaotic map $x(n+1)=a_{\left(0\right)}{\left[x(n)\right]}^{k}+a_{\left(1\right)}{\left[x(n)\right]}^{k-1}+\ldots +a_{\left(k-1\right)}x(n)+a_{\left(k\right)}$, k≥3.

In this section, we illustrate the proposed method by constructing a seven-degree polynomial chaotic map. Give different numbers for A=1, B=2, D=3, and let $a_{\left(0\right)}=a_{\left(1\right)}=a_{\left(2\right)}=a_{\left(3\right)}=a_{\left(4\right)}=0.0009$. We can obtain the coefficients $a_{\left(0\right)}=a_{\left(1\right)}=a_{\left(2\right)}=a_{\left(3\right)}=a_{\left(4\right)}=0.0009$, $a_{\left(5\right)}=-1.6388$, $a_{\left(6\right)}=5.8921$, and $a_{\left(7\right)}=-2.2538$. The reconstructed new seven-degree polynomial chaotic map is:

(31) $$x(n+1)=0.0009({\left[x(n)\right]}^{7}+{\left[x(n)\right]}^{6}+{\left[x(n)\right]}^{5}+{\left[x(n)\right]}^{4}+{\left[x(n)\right]}^{3})-1.6388{\left[x(n)\right]}^{2}+5.8921x(n)-2.2538$$

**Figure 4:**
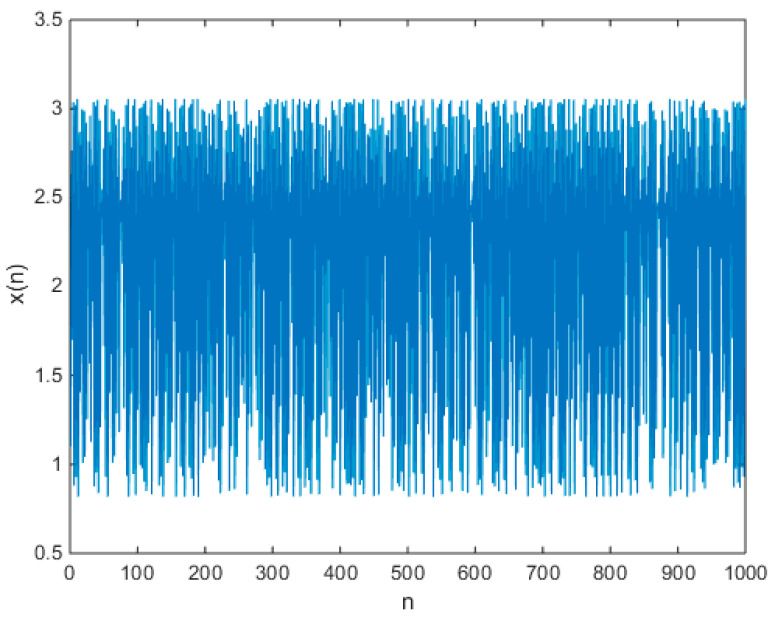
The output chaotic time series of the chaotic map (31).

The initial values of all the chaotic maps in Equation (17) are set as 1.1, and the chaotic time series are shown in Figure 4. Let m=2, N=1000, and r=0.15SD(x)=0.1174, the approximate entropy of chaotic map (31) is 0.4663.

## 5. Conclusions

In this paper, the period-three theorem was applied to the chaotization of discrete dynamical systems. Compared with other methods, the new method proposed in this paper was more effective for finding new quadratic polynomial chaotic maps and could accurately control the amplitude of new chaotic maps and existing of classical chaotic maps. After the analysis of existence and stability of fixed points, the quadratic polynomial maps with positive period-three points cannot have hidden chaotic attractors without fixed points or excited from stable fixed points.

## Figures and Tables

**Table 1:** Approximate entropy test.

Chaotic Map	m	r=0.15SD(x)	N	ApEn
$x(n+1)=-1.5{\left[x(n)\right]}^{2}+5.5x(n)-2$ (Equation (11))	2	0.1155	1000	0.4278
$x(n+1)=-3{\left[x(n)\right]}^{2}+3.46x(n)+0.18$ (Equation (15a))	2	0.0578	1000	0.3904
$x(n+1)=-3{\left[x(n)\right]}^{2}+6.34x(n)-1.69$ (Equation (15b))	2	0.0577	1000	0.3844
$x(n+1)=-0.3{\left[x(n)\right]}^{2}+2.6x(n)+4.75$ (Equation (15c))	2	0.5649	1000	0.4650
$x(n+1)=-150{\left[x(n)\right]}^{2}+194.5x(n)-62.39$ (Equation (15d))	2	0.0012	1000	0.4199
$x(n+1)=4x(n)-4{\left[x(n)\right]}^{2}$ (Equation (16))	2	0.0525	1000	0.6349
$x(n+1)=4x(n)-{\left[x(n)\right]}^{2}$ (Equation (17a))	2	0.2113	1000	0.6401
$x(n+1)=4x(n)-2{\left[x(n)\right]}^{2}$ (Equation (17b))	2	0.1056	1000	0.6333
$x(n+1)=4x(n)-8{\left[x(n)\right]}^{2}$ (Equation (17c))	2	0.0262	1000	0.6353
$x(n+1)=4x(n)-16{\left[x(n)\right]}^{2}$ (Equation (17d))	2	0.0132	1000	0.6508
